# α-Smooth Muscle Actin as Predictors of Early Recurrence in Early-Stage Ductal Type Breast Cancer After Mastectomy and Chemotherapy

**DOI:** 10.30699/IJP.2023.2004468.3126

**Published:** 2023-12-29

**Authors:** Andreas Andrianto, I. Ketut Sudiana, Desak G. A. Suprabawati

**Affiliations:** 1 *Doctoral Program of Medical Science, Faculty of Medicine, Universitas Airlangga, Surabaya, Indonesia*; 2 *Department of Pathology Anatomy, Faculty of Medicine, Universitas Airlangga, Surabaya, Indonesia*; 3 *Division of Oncology, Department of Surgery, Faculty of Medicine, Universitas Airlangga, Surabaya, Indonesia*

**Keywords:** Early-stage IDC, Recurrence predictor, α-SMA

## Abstract

**Background & Objective:**

Breast cancer recurrence after surgery was a sign that the progress of the disease was continuing. Early detection of breast cancer patients who are at risk requires development of a marker. Alfa smooth muscle actin (α-SMA) plays a role in the local recurrence process of invasive ductal carcinoma (IDC). Currently, existing tumor markers are used to predict the prognosis of breast cancer in general, not the early stages. Therefore, it was thought that finding α-SMA expression might predict early recurrence in early-stage IDC more accurately than others. This study investigated the potential role of α-SMA expression as a predictor of early recurrence in early-stage IDC and its relationship to clinicopathological factors.

**Methods:**

The study design was cross-sectional, with data obtained from the medical records of Dr. Koesnadi, General Hospital, Bondowoso, Indonesia. Bivariate and multivariate analysis was performed to analyze data.

**Results:**

We included 50 subjects divided into the local recurrence group (n=25) and the non-local recurrence group (n=25). We found a statistically significant correlation between the incidence of local recurrence in early-stage IDC and the high expression of α-SMA (odd ratio [OR]=23.22, 95% confidence interval [CI]=5.101-105.7, *P*=0.001). Clinicopathological variables and α-SMA expression did not have a significant correlation.

**Conclusion:**

In early-stage IDC, α-SMA expression had the potential to predict and could be an independent prognostic factor for early recurrence.

## Introduction

The prognosis for early-stage breast cancer that receives adequate treatment is favorable. Recurrence following adequate treatment will worsen the prognosis. The five-year survival rate for patients who encounter recurrence is 35% to 59%, similar to that for patients with breast cancer who have developed distant metastasis (1). The most critical period for recurrence is the first two years after surgery (2). Adjuvant chemotherapy is one of the methods used to lower the risk of recurrence. In early-stage breast cancer, adjuvant chemotherapy reduces the likelihood of micrometastasis. Adjuvant chemotherapy can improve disease-free survival and overall survival in patients with early-stage breast cancer, according to a study by the Early Breast Cancer Trialists Collaborative Group (EBCTG) (3). A multidrug chemotherapy regimen is typically used to increase treatment efficacy and decrease adverse effects. Combining anthracycline and taxane groups is the first-choice combination of chemotherapy medications for breast cancer(4).

Breast cancer remains a significant global health concern and is the leading cause of death from cancer in women (5). The two most prevalent histological forms are invasive ductal carcinoma (IDC), with a frequency of 80%, and invasive lobular carcinoma (ILC), with a frequency of 15% (6). Only 20–30% of breast cancer patients are diagnosed early, whereas 50–70% are diagnosed at advanced stages (7). Breast-conserving surgery (BCS) and Modified Radical Mastectomy or Modified Radical Mastectomy (MRM) are two of the most common surgical treatments for breast cancer. Breast-conserving surgery consists of an extensive excision of the tumor and procedures in the ipsilateral axilla (level I and II axillary dissections or sentinel node biopsy). Modified radical mastectomy is a procedure that removes the tumor, the skin over the tumor, all breast glands, and the axilla concurrently with axillary dissection. Even though the entire breast has been removed in the mastectomy surgery, recurrence can still occur. In patients with a high risk of recurrence, adjuvant chemotherapy is usually given to reduce the possibility of recurrence.

Breast cancer recurrence can occur if cancer cells can evade the body's immune system. Cancer cells must be able to avoid apoptosis by producing proteins to protect themselves and altering their signaling pathways (8). Mechanisms to avoid apoptosis include alteration of the signaling and transport systems in the cell membrane (including membrane receptors), modifications to the DNA repair process (cancer stemness), and modifications to the tumor microenvironment (niche) (9). Cancer cells will change normal fibroblast cells into cancer-associated fibroblasts (CAF) so that the microenvironment is more conducive to survival and dissemination (metastasis). CAF modifies the structure and form of normal fibroblasts to facilitate the metastatic process. Cancer cells and fibroblasts are constantly in contact, either directly through cell-to-cell contact or through the production of soluble substances that can activate several signaling pathways in tumor cells.

The connective tissue contains fibroblasts, neither vascular, epithelial, nor inflammatory cells. It is well known that fibroblasts comprise a sizeable portion of the stroma in various malignancies, and roughly 80% of stromal fibroblasts in breast carcinomas develop a changed phenotype (10). These activated fibroblasts, also known as myofibroblasts or carcinoma-associated fibroblasts, are morphologically distinguished by giant spindle-shaped cells with smooth muscle phenotypic features and are frequently recognized by the expression of α-smooth-muscle actin (α-SMA) in malignancy. The myofibroblast is a cell that possesses the rough endoplasmic reticulum and Golgi complex of fibroblasts and the bundles of myofilaments with thick bodies found in smooth muscle cells. A significant cytoplasmic microfilamentous apparatus and the term "myofibroblast" had been suggested for fibroblastic cells within granulation tissue a few decades ago. Breast cancer cells can transformed myoepithelial cells develop characteristic features of myofibroblasts: bundles of intracytoplasmic microfilament; abundant rough endoplasmic reticulum; prominent Golgi complex; and surface membrane differentiations that provide attachment to neighboring epithelial cells through epithelial to mesenchymal transition (EMT) (11).

In contrast to normal fibroblasts, which remodel the extracellular matrix (ECM), regulate normal tissue homeostasis, and participate in wound healing and senescence, CAFs can promote tumorigenesis. Multiple diverse functions of CAFs in cancer are extracellular matrix remodeling, maintenance of stemness, blood vessel formation, and promotion of cancer cell proliferation, migration, and invasion, all of which contribute to therapy resistance and the formation of metastases, which CAFs mediate. Multiple studies in recent years have demonstrated that CAFs play a crucial role in transforming the metabolic landscape of tumors. Moreover, CAFs can regulate the neighboring immune cells that contribute to tumor immune evasion via multiple mechanisms, such as the secretion of multiple cytokines and chemokines and the recruitment and modulation of tumor-infiltrating immune cells. Interactions between CAFs and cancer cells are mediated by intracellular and extracellular factors that could potentially be targeted by anticancer therapies (12). The increased expression of α-SMA identifies the formation of CAFs (13). Increased α-SMA expression correlates with the incidence of EMT (13, 14) and lymph node metastases, resulting in a poorer prognosis (14). EMT is the process by which epithelial cells transform into mesenchymal cells, rendering them more aggressive, prone to metastasis, and resistant to anthracycline chemotherapy (15). Mesenchymal cell tissue is less compact and more diffuse than epithelial cell tissue. The connections between mesenchymal cells are only present on one side of the cell, not both.

Contrary to epithelial cells, which exclusively have basal lamina-bound ECM, ECM is dispersed across the cell surface of mesenchymal cells. Mesenchymal cells lack a distinct polarity and are arranged in looser bonds, making them more flexible, mobile, and individualized (16). The expression of α-SMA as a predictor of local recurrence within two years following mastectomy in early-stage IDC has not yet been studied. This study aimed to examine whether the expression of α-SMA could predict early local recurrence in early-stage IDC and investigate the relationship between α-SMA and clinicopathological variables.

## Material and Methods

**Study Design and Subjects**

This research was an analytic observational study with a cross-sectional design. The research data were obtained from medical records in the surgical department of Dr. H Koesnadi Bondowoso General Hospital, East Java, Indonesia (RSDK). The study was conducted following the Declaration of Helsinki and approved by The Research Ethics Committee of the Faculty of Medicine, State University of Jember, with number 1.537/H25.1.11/KE/2021. The breast cancer diagnosis was based on the pathology report of the surgical specimen. Two anatomical pathology specialists carried out all histopathological examinations. The study subjects were early-stage IDC patients who underwent MRM performed by the first author at RSDK from January 2014 - December 2019 (5 years). The sample size was 25 for the group with local recurrence and 25 without recurrence. The decision number of the sample size is based on the minimum number of samples needed for regression analysis (17). The inclusion criteria for this study were patients with early-stage (stadium I and II) IDC who underwent MRM procedures and received one cycle adjuvant chemotherapy of the taxane and anthracycline-based regimen. Adjuvant chemotherapy with taxane and anthracycline base regimens was given six times every three weeks (one cycle). The exclusion criteria of this study were: patients received external radiation therapy, another malignancy in other organs, pathology examination report stating that the edge of the resection was not tumor-free (less than two centimeters), paraffin blocks were damaged and could not be used. The stages of cancer were obtained from clinical examination, radiology, and pathology reports. Pathological grading was evaluated by an anatomic pathologist based on the Bloom-Richardson grading system (18). After receiving an explanation, all patients who participated in this study signed a consent form to participate and a consent form for publication. If the patient has died, the consent form is signed by the child or the patient's next of kin.

**Immunohistochemistry (IHC) Staining of** **α** **-SMA**

Block paraffin for immunohistochemistry was obtained from MRM surgery specimens. Each 4 m tissue block embedded in paraffin was deposited on an object glass and heated at 600°C for 60 minutes. Each section was deparaffinized three times for three minutes using xylene. Then, each section was rehydrated with ethanol serial dilutions (100%, 96%, and 70%) for 3 minutes, followed by 3 minutes of water cleansing. Thirty minutes of endogen peroxidase blocking with 0.5% H_2_O_2_ in methanol was followed by 5 minutes of water purification. In a 96°C decloaking chamber with a pH 9.0 Tris-EDTA (TE) solution, a 10-minute antigen retrieval procedure was used to perform the pretreatment. Then, a 10-minute peroxidase block was performed. Before and after the peroxidase block, the sample was washed for three minutes with pH 7.4 Phosphate-buffer saline (PBS). The sniper background was blocked for 10 minutes and then rinsed with PBS for 3 minutes. In normal serum, the primary antibody against α-SMA was incubated for 60 minutes at a dilution of 1:50. This investigation utilized the α-SMA human monoclonal antibody from MyBioSource with catalog number MBS266274. Then, the subsequent phases were: Three minutes of PBS rinsing, thirty minutes of post-primary, and three minutes of PBS washing with polymer in thirty minutes, five minutes of PBS washing, one to two minutes of 3,3'-Diaminobenzidine (DAB), and two minutes of water cleansing. Counterstaining with CAT hematoxylin was conducted for ten seconds. The sample was immersed for 5 seconds in lithium carbonate (5% in aquadest) to produce a blue nucleus. Each section was dehydrated in ascending ethanol concentrations for 3 minutes, followed by xylene clearance and coverslipping.

**IHC Staining Assessment**

Expression of α-SMA was assessed by looking at the staining intensity of the tumor cell's cytoplasm and counting the number of positive tumor cells. Ten high-power fields (400x) were randomly selected and analyzed for each sample, and then the number of positive cells was averaged.

**Statistical Analysis**

The Openepi 3.0 programs and EZR were used to process the data. The Chi-square test and Fisher's exact test were performed in the bivariate analysis process. The sensitivity and specificity values acquired from the ROC graph determine the cut-off points for the α-SMA expressions. Logistic regression tests are used to conduct multivariate clinical and pathological characteristics analyses. A statistical value of 0.05 or less is regarded to be significant.

## Results

**Characteristics of the Patients**

In this study, there were 50 subjects, including 25 subjects with no recurrence and 25 subjects with recurrence. According to the findings of the bivariate analysis, there were no significant differences between the two groups in terms of clinical and pathological data. Table 1 provides information on the outcomes of the bivariate analysis that was carried out.

**Table 1:** The relationship between the characteristics of the study sample and local recurrence

	Recurrence (-) n=25)	Recurrence (+) (n=25)	*P*
Age (mean)	50.25±14.93	51.00±9.57	0.920
KGB metastases (mean)	1.25±1.5	3.00±1.53	0.09
Grade (mean)	1.86±0.00	2±0.69	0.61
Hormonal status			
Pre menopause	1	5	0.197
Menopause	3	2	-
α-SMA expression (mean)	3.09±1.37	5.59±1,85	< 0.000

**Analysis of α-SMA Expression and Local Recurrence in Early Stage IDC**

**Fig. 1:**
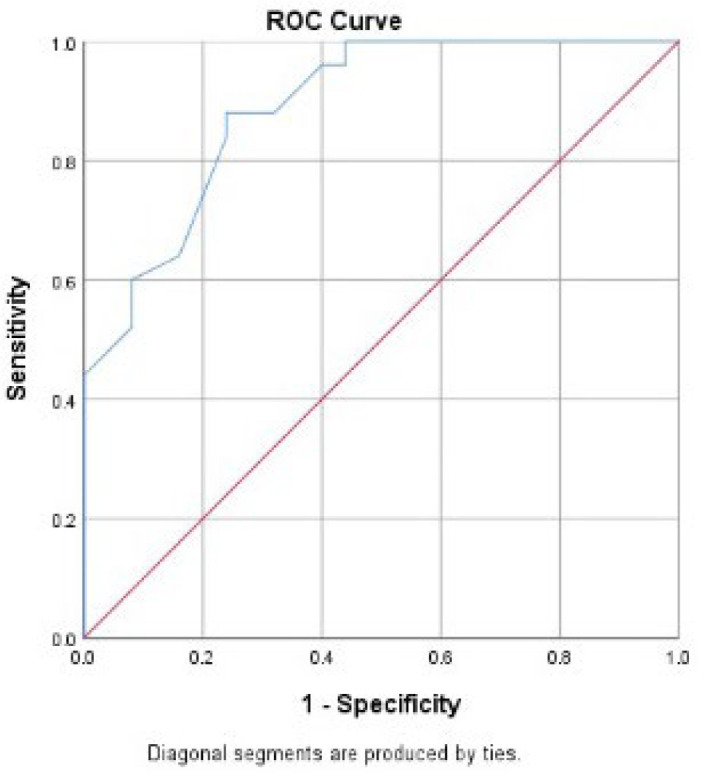
ROC curve of expression of α-SMA in early-stage IDC

**Fig. 2:**
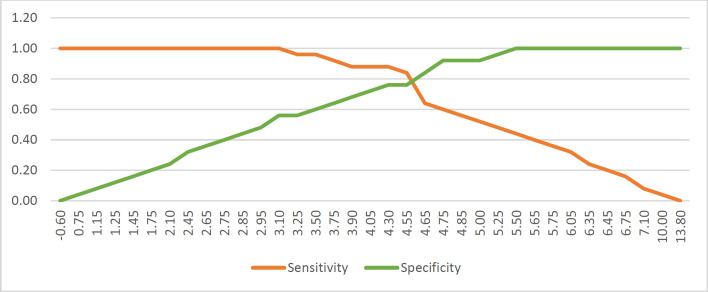
Sensitivity and specificity of α-SMA examination. The *cut-off* value used is 4.3

Data from each sample was calculated to determine the H-score. The ROC curve is provided in Figure 1. The cut-off point was defined using the sensitivity and specificity graph shown in Figure 2. The defined cut-off value for the H-score was 4.3; we consider the α-SMA expression to be high if the H-score value is≥ 4.3 and low if the H-score value is < 4.3.

**Association Between α-SMA Expression and Local Recurrence in Early Stage IDC**

There was a statistically significant difference between the mean H-Score values of α-SMA expression in the non-local recurrence group (3.09±1.37) and the local recurrence group (5.59±1.85). The magnitude of the difference was 3.336, with a 95% CI of 2.754– 3.918. The Chi-Square test revealed a significant correlation between α-SMA expression and local recurrence in early-stage IDC patients (*P*<0.001), as presented in Table 2. Patients with breast cancer with high α-SMA expression were 23 times more likely to develop local recurrence than those with low α-SMA expression. Breast cancer patients with high α-SMA expression were 23 times more likely to develop local recurrence than those with low expression of α-SMA. Table 4 provides the calculated sensitivity, specificity, positive predictive value, negative predictive value, positive likelihood ratio, negative likelihood ratio, and accuracy of α-SMA expression for predicting local recurrence in IDC patients. This calculation is founded on the findings of this study, as presented in Table 2.

**Association Between the Clinicopathological Factors and α-SMA Expression**

The bivariate analysis revealed no statistically significant relationship between clinicopathological factors and α-SMA expression. In a multivariate analysis between clinicopathological factors and the expression of α-SMA, factors with a P-value <0.25 in the preceding bivariate analysis were included; this included age and metastatic lymph nodes. Multivariate analysis revealed no significant associations between factors and α-SMA expression (*P*>0.05). Table 3 provides information on bivariate and multivariate analysis of the association between clinicopathological factors and α-SMA expression.

**Table 2:** Relationship between α-SMA expression and local recurrence

Type		Recurrent		OR(95%CI)	*P*
		(+)	(-)	23.22 (5.101-105.7)	<0.000
α-SMA expression	High	22	6		
	Low	3	19		

**Table 3:** Analysis of the bivariate and multivariate relationship between clinicopathological factors and α-SMA expression

Variable	Category	Bivariate Analysis (α-SMA)		*P*	Multivariate analysis OR(95%CI)*	*P*
Mean Age (years)		Low (n=22)	High (n=28)			
		52.23±11.1	49.75±11.73	0.452	NA	0.444
Age category	≤40	1	8	0.06	0.612(0.175-2.148)	
	>40	21	2			
Lymphnode metastasis	≤3	13	21	0.231	0.133(0.015-1.188)	0.071
	>3	9	7			
Grade	Low	4	4	0.718	NA	
	High	18	24			
Menopause	Pre	11	15	0.802	NA	
	Post	11	13			

**Table 4:** Diagnostic indicators of α-SMA for local recurrence in early-stage IDC

Indicators	Value
Sensitivity	88%
Specificity	76%
Positive predictive value	78,6%
Negative predictive value	86,4%
Positive likelihood ratio	3.67
Negative likelihood ratio	0.16
Accuracy	82%

## Discussion

**Patients Characteristic**

In this study, the average age of patients who did not get local recurrence was 50.25±14.93 and 51.00±9.57 in the groups who got local recurrence. The patient's age at the diagnosis was considered one of the most important prognostic markers in breast cancer. Breast cancer at a younger age tends to be more aggressive and resistant to hormonal therapy. According to the findings of several epidemiological research, people diagnosed with breast cancer at a younger age had a worse prognosis than those diagnosed at an older age (19). There is a strong correlation between mutations in the BRCA1/2 gene and the development of breast cancer in younger women. Another important prognostic factor for breast cancer was ipsilateral lymph node metastasis. The number of lymph nodes in which breast cancer has metastasis is directly proportional to the patient's prognosis (20). The involvement of regional lymph nodes is another helpful characteristic that can be used to evaluate the likelihood of a patient's breast cancer local recurrence and their likelihood of survival. The research conducted by Shen *et al.* showed that the prognosis of invasive breast cancer was correlated not to the level at which the metastases appeared but rather to the number of lymph nodes that had metastasis (21). Tumor grades indicate how aggressive the tumor was, and it correlates with metastasis and recurrence. According to the findings of studies that Bijker carried out, there is a correlation between the tumor grade and breast cancer recurrence after breast BCS (22). In a prospective study in Thailand, Auayporn found a similar result: high-grade tumors tend to recurrence (OR=2.9) compared to low-grade tumors (23). Another factor that also affects the patient's prognosis is menopausal status. Breast cancer in premenopausal women tends to be resistant to chemotherapy compared to post-menopausal women. This study showed that there was no significant difference between the non-recurrence group and the recurrence group. The results of non-parametric statistical tests, bivariate tests, and multivariate tests for clinicopathological factors such as age, lymph node metastases, tumor grade, and menopausal status showed no significant differences (*P*>0.05) in this study. These results are different from other studies because it may be because the samples in this study were more homogeneous, invasive ductal carcinoma. The tumor sizes in this study were all T1-2, so they were more homogeneous in size and grading. Research conducted by Vicini states that in early-stage breast cancer, lymph node metastases do not significantly affect prognosis (24).

Cancer cells can communicate with other cells through paracrine signaling pathways. Cancer cells will reshape the surrounding microenvironment through paracrine signaling to protect themselves from the body's defense mechanisms and unfavorable conditions, such as when administering chemotherapy drugs. The tumor microenvironment is an important driver of tumor growth and response to treatment, so novel treatments must be developed to target it. In tumor growth, angiogenesis, and progression, stromal cells are essential. For example, when exposed to paracrine signals triggered by malignant tumors, fibroblasts are mesenchymal cells that generally construct, maintain, and modify connective tissues; normal fibroblasts will differentiate into activated CAF. CAFs constitute the main population of tumor stroma. These cells are characterized by high α-SMA expression and excrete a panel of cytokines that act upon both stromal and tumor cells, amplifying both cell numbers and enhancing the malignant phenotype (25).

The cancer cells could change their natures to survive and metastasize through epithelial-to-mesenchymal transition (EMT). EMT is a process of changing the characteristics of epithelial to mesenchymal so that these cells have more aggressive, more frequent metastases and are resistant to anthracycline chemotherapy. Cancer cells that have undergone the EMT process are able to influence many signaling pathways, both intracellular and extracellular. One pathway that is also affected is the Platelet Derived Growth Factor (PDGF) pathway. Currently, there are four known PDGFs: PDGFA, PDGF-B, PDGF-C, and PDGF-D. Platelet-derived growth Factor-D (PDGF-D) regulates EMT and oncogenic activity by regulating cell growth, invasion, and metastasis (26). PDGF is also a regulator of lamellipodia formation, making tumor cells more aggressive and resistant to treatment (27). In order to support the development of metastasis, CAF changes the structure and shape of fibroblasts. A higher expression of α-SMA can identify the formation of CAF (28). Studies conducted by Damonte showed that increased expression of α-SMA also correlated with the incidence of EMT (29). As a main component of the tumor stroma, CAF is crucial in the progression of many solid tumors through communication with cancer cells. Several oncological functions of CAFs have been identified as promoting tumor progression. Gaggioli *et al.* demonstrated, for instance, that CAFs are necessary to facilitate the invasion of squamous cell carcinoma cells (30). CAFs promote the chemoresistance of cancer cells in pancreatic cancer via signaling mediated by exosomes. Additionally, CAFs have been shown to promote tumor growth and angiogenesis in invasive breast carcinomas (31). Yang *et al.* stated that CAF regulates apoptosis to promote cancer dissemination in colorectal cancer cells (32). 

There were six subjects with high expressions of α-SMA and 19 subjects with low expression in the group without local recurrence, whereas, in the group with local recurrence, we found high expressions of α-SMA in 22 subjects and low expressions of α-SMA in 3 subjects. Statistical tests with the Chi-square test showed an Odd Ratio (95% CI) of 23.22 (5.101-105.7). Hence, breast cancer patients with high α-SMA expression are more likely to have a local recurrence than patients with low α-SMA expression. Research conducted by Zhan also shows that high α-sma expression correlates with a worse prognosis and recurrence of gastric cancer (33). A study by Qiao showed that over-expression of CAF also correlated with chemoresistance in oesophageal cancer (34). A study by Amornsupak also found that CAF expression was associated with resistance to the chemotherapy drug doxorubicin (taxane) in breast cancer (35). In this study, the sensitivity of the α-SMA diagnostic test for local recurrence was 88%, the specificity was 76%, the positive predictive value was 78.6%, the negative predictive value was 86.4%, the positive likelihood ratio was 3.67, the negative likelihood ratio was 0.16, and the accuracy was 82%. Therefore, detecting α-SMA expression can be used in breast cancer patient screening and as a predictor of local recurrence. Our findings are similar to other biomarker predictors for breast cancer recurrence. A study by Ojha on predicting breast cancer recurrence using data mining techniques with Support Vector Machines (SVM) showed that currently established biomarkers have the highest accuracy of 1% (36). In another study, Zain *et al.* predicted the recurrence of breast cancer using principal component analysis (PCA) techniques; it was found that the current biomarkers have an accuracy of 76.1% (37). Based on these findings, we concluded that α-SMA expression can be used as a predictor of local recurrence in breast cancer. Ultimately, the authors recognize that the study has limitations despite our homogeneous research population. This study was a cross-sectional study with a small sample size and a brief duration of follow-up. Conducting a future inves-tigation with a larger sample size would be more advantageous.

## Conclusion

Early local recurrence in early-stage IDC can be predicted by α-SMA expression. Despite the low specificity of this predictor, this study may expand clinicians' understanding of α-SMA expression test usage in breast cancer patients to other diagnostic modalities.

## Conflict of Interest

All the authors declared no conflict of interest.

## References

[1] Ren' R, Mulder RL, Hudson MM, Bhatia S, Landier W, Levitt ; Gill (2020). Updated Breast Cancer Surveillance Recommendations for Female Survivors of Childhood, Adolescent, and Young Adult Cancer From the International Guideline Harmonization Group. J Clin Oncol..

[2] Wapnir IL, Anderson SJ, Mamounas EP, Geyer CE, Jeong JH, Tan-Chiu E (2006). Prognosis after ipsilateral breast tumor recurrence and locoregional recurrences in five national surgical adjuvant breast and bowel project node-positive adjuvant breast cancer trials. J Clin Oncol.

[3] Davies C, Godwin J, Gray R, Clarke M, Cutter D, Darby S (2011). Early Breast Cancer Trialists' Collaborative Group (EBCTCG) Relevance of breast cancer hormone receptors and other factors to the efficacy of adjuvant tamoxifen: Patient-level meta-analysis of randomised trials. Lancet.

[4] Taghian AG, Jeong JH, Mamounas EP, Parda DS, Deutsch M, Costantino JP (2006). Low locoregional recurrence rate among node-negative breast cancer patients with tumors 5 cm or larger treated by mastectomy, with or without adjuvant systemic therapy and without radiotherapy: Results from five National Surgical Adjuvant Breast and Bowel. J Clin Oncol.

[5] Sung H, Ferlay J, Siegel RL, Laversanne M, Soerjomataram I, Jemal A (2021). Global Cancer Statistics 2020: GLOBOCAN Estimates of Incidence and Mortality Worldwide for 36 Cancers in 185 Countries. CA Cancer J Clin.

[6] Globocan (2018 ). Cancer Today [Internet].

[7] Panigoro SonarS, Karsono R, Sari L (2017). E-cadherin and Vimentin as Predictors of Resistance to Preoperative Systemic Therapy in Patients with Advanced Breast Cancer. J Kedokteran Indonesia.

[8] Fernald K, Kurokawa M (2013). Evading apoptosis in cancer. Trends Cell Biol.

[9] Igney FH, Krammer PH (2002). Immune escape of tumors: apoptosis resistance and tumor counterattack. J Leukoc Biol.

[10] Mangia A, Malfettone A, Rossi R, Paradiso A, Ranieri G, Simone G (2011). Tissue remodelling in breast cancer: human mast cell tryptase as an initiator of myofibroblast differentiation. Histopathology.

[11] Gabbiani G (1998). Evolution and clinical implications of the myofibroblast concept. Cardiovasc Res.

[12] Fernández-Nogueira P, Fuster G, Gutierrez-Uzquiza Á, Gascón P, Carbó N, Bragado P (2021). Cancer-Associated Fibroblasts in Breast Cancer Treatment Response and Metastasis. Cacers.

[13] Tang X, Tu G, Yang G, Wang X, Kang L, Yang L (2019). Autocrine TGF-β1/miR-200s/miR-221/DNMT3B regulatory loop maintains CAF status to fuel breast cancer cell proliferation. Cancer Lett.

[14] Bill R, Christofori G (2015). The relevance of EMT in breast cancer metastasis: Correlation or causality?. FEBS Lett.

[15] Beerling E, Seinstra D, de Wit E, Kester L, van der Velden D, Maynard C (2016). Plasticity between Epithelial and Mesenchymal States Unlinks EMT from Metastasis-Enhancing Stem Cell Capacity. Cell Rep.

[16] Panawala L (2018). Difference Between Epithelial and Mesenchymal Cells Main Difference - Epithelial vs Mesenchymal Cells.

[17] Kanda Y (2013). Investigation of the freely available easy-to-use software "EZR" for medical statistics. Bone Marrow Transplant.

[18] Rui Z (2011). Reproducibility of the Notingham modification of the Scarf- Bloom-Richards on histological grading system and the com plem entary value ofK i一67 to this system. Chin Med J.

[19] Gnerlich JL, Deshpande AD, Jeffe DB, Sweet A, White N, Margenthaler JA (2009). Elevated Breast Cancer Mortality in Women Younger than Age 40 Years Compared with Older Women Is Attributed to Poorer Survival in Early-Stage Disease. J Am Coll Surg.

[20] Galea MH, Blarney RW, Elston CE, Ellis IO (1992). The Nottingham Prognostic Index in primary breast cancer. Breast Cancer Res Treat..

[21] Shen ZZ (1991 ). Relation of tumor size, lymph node status and prognosis in breast cancer. Chin J Surg.

[22] Bijker N, Meijnen P, Peterse JL, Bogaerts J, Van Hoorebeeck I, Julien JP (2006). Breast-conserving treatment with or without radiotherapy in ductal carcinoma-in-situ: Ten-year results of european organisation for research and treatment of cancer randomized phase III trial 10853 - A study by the EORTC breast cancer cooperative group an. J Clin Oncol.

[23] Auayporn M (2009). Exploring Breast Cancer Data to Find the Risk Factors for Distant Metastasis: Register Data, The National Cancer Institute of Thailand Montida Auayporn Piangchan Rojanavipart Chukiat Viwatwongkasem Arkom Chaiwerawattana Kitiphong Hancharoen. J Public Health.

[24] Vicini FA, Kestin L, Huang R, Martinez A (2003). Does local recurrence affect the rate of distant metastases and survival in patients with early-stage breast carcinoma treated with breast-conserving therapy?. Cancer.

[25] Murakami M, Ernsting MJ, Undzys E, Holwell N, Foltz WD, Li SD (2013). Docetaxel conjugate nanoparticles that target a-smooth muscle actin-expressing stromal cells suppress breast cancer metastasis. Cancer Res.

[26] Singh A, Settleman JE (2010). EMT, cancer stem cells and drug resistance: an emerging axis of evil in the war on cancer. Oncogene.

[27] Paulsson J, Ryd L, Strell C, Frings O, Tobin NP, Fornander T (2017). High expression of stromal PDGFR b is associated with reduced benefit of tamoxifen in breast cancer. J Pathol Clin Res J..

[28] Lazard D, Sastre X, Frid MG, Glukhova MA, Thiery JP, Koteliansky V (1993). Expression of smooth muscle-specific proteins in myoepithelium and stromal myofibroblasts of normal and malignant human breast tissue. Proceedings of the National Academy of Sciences.

[29] Damonte P, Gregg JP, Borowsky AD, Keister BA, Cardiff RD (2007). EMT tumorigenesis in the mouse mammary gland. Lab Invest.

[30] Gaggioli C, Hooper S, Hidalgo-Carcedo C, Grosse R, Marshall JF, Harrington K (2007). Fibroblast-led collective invasion of carcinoma cells with differing roles for RhoGTPases in leading and following cells. Nat Cell Biol.

[31] Orimo A, Gupta PB, Sgroi DC, Arenzana-Seisdedos F, Delaunay T, Naeem R (2005). Stromal fibroblasts present in invasive human breast carcinomas promote tumor growth and angiogenesis through elevated SDF-1/CXCL12 secretion. Cell.

[32] Yang K, Zhang J, Bao C (2021). Exosomal circEIF3K from cancer-associated fibroblast promotes colorectal cancer (CRC) progression via miR-214/PD-L1 axis. BMC Cancer.

[33] Zhan S, Liu Z, Zhang M, Guo T, Quan Q, Huang L (2020). Overexpression of B7-H3 in α-SMA-Positive Fibroblasts Is Associated With Cancer Progression and Survival in Gastric Adenocarcinomas. Front Oncol.

[34] Qiao Y, Zhang C, Li A, Wang D, Luo Z, Ping Y (2018). IL6 derived from cancer-associated fibroblasts promotes chemoresistance via CXCR7 in esophageal squamous cell carcinoma. Oncogene.

[35] Amornsupak K, Insawang T, Thuwajit P, O-Charoenrat P, Eccles SA, Thuwajit C (2014). Cancer-associated fibroblasts induce high mobility group box 1 and contribute to resistance to doxorubicin in breast cancer cells. BMC Cancer.

[36] Ojha U, Goel S (2017). A study on prediction of breast cancer recurrence using data mining techniques.

[37] Zain ZM, Alshenaifi M, Aljaloud A, Albednah T, Alghanim R, Alqifari A (2020). Predicting breast cancer recurrence using principal component analysis as feature extraction: An unbiased comparative analysis. Int J Adv Intell Informatics.

